# COVID-19 vaccination effectiveness and safety in vulnerable populations: a meta-analysis of 33 observational studies

**DOI:** 10.3389/fphar.2023.1144824

**Published:** 2023-06-23

**Authors:** Hui-Jun Li, Qi-Chao Yang, Yang-Yang Yao, Cheng-Yang Huang, Fu-Qiang Yin, Chen-Yang Xian-Yu, Chao Zhang, Shao-Juan Chen

**Affiliations:** ^1^ Center for Evidence-Based Medicine and Clinical Research, Taihe Hospital, Hubei University of Medicine, Shiyan, Hubei, China; ^2^ Department of Emergency, Renmin Hospital, Hubei University of Medicine, Shiyan, Hubei, China; ^3^ Department of Stomatology, Taihe Hospital, Hubei University of Medicine, Shiyan, Hubei, China

**Keywords:** vulnerable populations, COVID-19 vaccination, haematological cancers, organ transplant, elderly, meta-analysis

## Abstract

**Background:** Even 3 years into the COVID-19 pandemic, questions remain about how to safely and effectively vaccinate vulnerable populations. A systematic analysis of the safety and efficacy of the COVID-19 vaccine in at-risk groups has not been conducted to date.

**Methods:** This study involved a comprehensive search of PubMed, EMBASE, and Cochrane Central Controlled Trial Registry data through 12 July 2022. Post-vaccination outcomes included the number of humoral and cellular immune responders in vulnerable and healthy populations, antibody levels in humoral immune responders, and adverse events.

**Results:** A total of 23 articles assessing 32 studies, were included. The levels of IgG (SMD = −1.82, 95% CI [−2.28, −1.35]), IgA (SMD = −0.37, 95% CI [−0.70, −0.03]), IgM (SMD = −0.94, 95% CI [−1.38, −0.51]), neutralizing antibodies (SMD = −1.37, 95% CI [−2.62, −0.11]), and T cells (SMD = −1.98, 95% CI [−3.44, −0.53]) were significantly lower in vulnerable than in healthy populations. The positive detection rates of IgG (OR = 0.05, 95% CI [0.02, 0.14]) and IgA (OR = 0.03, 95% CI [0.01, 0.11]) antibodies and the cellular immune response rates (OR = 0.20, 95% CI [0.09, 0.45]) were also lower in the vulnerable populations. There were no statistically significant differences in fever (OR = 2.53, 95% CI [0.11, 60.86]), chills (OR = 2.03, 95% CI [0.08, 53.85]), myalgia (OR = 10.31, 95% CI [0.56, 191.08]), local pain at the injection site (OR = 17.83, 95% CI [0.32, 989.06]), headache (OR = 53.57, 95% CI [3.21, 892.79]), tenderness (OR = 2.68, 95% CI [0.49, 14.73]), and fatigue (OR = 22.89, 95% CI [0.45, 1164.22]) between the vulnerable and healthy populations.

**Conclusion:** Seroconversion rates after COVID-19 vaccination were generally worse in the vulnerable than healthy populations, but there was no difference in adverse events. Patients with hematological cancers had the lowest IgG antibody levels of all the vulnerable populations, so closer attention to these patients is recommended. Subjects who received the combined vaccine had higher antibody levels than those who received the single vaccine.

## 1 Introduction

Coronavirus disease 2019 (COVID-19), a highly transmissible viral illness caused by severe acute respiratory syndrome coronavirus 2 (SARS-CoV-2), quickly spread worldwide and became a significant global health crisis (Chan et al., 2020). While SARS-CoV-2 infection can have a debilitating long-term effect on daily activities, no drugs are available to safely and effectively treat COVID-19 (Huang et al., 2020). As a result, timely vaccination is the most effective preventive measure to minimize the negative impact of this infection at both the individual and community level (Majid et al., 2021).

Deployment of the COVID-19 vaccine cannot be standardized across the entire population because particular subgroups carry different risks of exposure to SARS-CoV-2 (Grassly et al., 2015; Koff and Williams, 2020). Some individuals are at higher risk of becoming infected and/or developing severe disease or may not have access to timely and effective treatment (Koff et al., 2021). Thus, strengthening prevention efforts among vulnerable populations, including ensuring adequate COVID-19 vaccination coverage, can help reduce the risk of a large-scale outbreak (Lipsitch and Dean, 2020). Populations considered vulnerable to COVID-19 include the elderly, pregnant women, children, people with underlying medical conditions, and those with limited access to primary medical care (Agrati et al., 2021).

The safest and most effective method of vaccinating vulnerable populations against COVID-19 remains unclear, and particular subgroups are still hesitant to receive the vaccine (Grassly et al., 2015). One study found that the infection rate of kidney transplant patients who received the same vaccination regimen as healthy patients at the peak of the pandemic remained unchanged, suggesting that this at-risk population requires an individualized vaccination protocol (Swai et al., 2022). Few studies have assessed the impact of COVID-19 vaccination in elderly populations, so evidence is lacking on how best to support the development of a vaccine initiative for this age group (Veronese et al., 2021). Seroconversion following COVID-19 vaccination is often lower in vulnerable than healthy populations (Thuluvath et al., 2021). Systematic analysis of available data is needed to explore options for increasing seroconversion and assessing the safety of COVID-19 vaccination of at-risk populations.

The current study evaluated humoral and cellular immune responses to the COVID-19 vaccine and the adverse effects (AEs) of vaccination in vulnerable and healthy populations. Meta-analysis and subgroup analyses were performed to comprehensively assess vaccine efficacy and safety in at-risk populations. The findings should inform the development of a protocol for COVID-19 vaccination of different vulnerable populations and help to alleviate vaccine hesitation.

## 2 Methods

This study was performed according to the Preferred Reporting Initiative for Systematic Assessment and Meta-Analysis (PRISMA) guidelines (Page et al., 2021).

### 2.1 Search strategy

This study conducted a meta-analysis of reports in PubMed, EMbase, and the Cochrane Central Register of Controlled Trials (CENTRAL) through 12 July 2022. Mesh terms including “vulnerable populations,” “chronic disease,” “underserved populations,” “sensitive populations,” “immunocompromised patients,” “aged,” “organ transplantation,” “neoplasms,” “tumor,” “cancer,” and “COVID-19 vaccines,” were used to search the three databases. A manual search of references from the included studies was also performed. The search strategies are described in Supplementary Method S1.

### 2.2 Inclusion and exclusion criteria

The subjects included in this meta-analysis were vulnerable and healthy recipients of the COVID-19 vaccine. The exposure group, defined as vulnerable, included elderly patients, immunocompromised patients, organ transplant recipients, and cancer patients. Elderly subjects were defined as those ≥60 years of age. Immunocompromised patients included AIDS patients, maintenance hemodialysis patients, and those receiving immunosuppressive drugs. Organ transplant recipients included common kidney and liver transplant patients. Cancer patients included those with solid cancers or hematological malignancies. The non-exposure group was defined as healthy. None of the subjects were previously infected with COVID-19. Outcomes included the number of subjects in each group with a measurable humoral and cellular immune response, the levels of antibodies in humoral immune responders after vaccination, and vaccination-related AEs [including fever, chills, myalgia, local pain at the site of injection, headache, tenderness, fatigue, gastrointestinal disturbances, arthralgia, local reddening, local swelling, lymph node swelling, malaise, flu-like symptoms, and a need for non-steroidal anti-inflammatory drugs (NSAIDs)]. Subjects with detectable serum levels of SARS-CoV-2 immunoglobulins (including IgG, IgA, and IgM) were defined as humoral immune responders. A measurable cellular immune response was defined as the presence of T cells that were activated in response to SARS-CoV-2 antigen. The primary outcomes included serum IgG antibody levels after vaccination and the number of virus-specific antibodies. Secondary outcomes included the number of patients with serum IgA and neutralizing antibody levels, IgA and IgM antibody levels, the cellular immune response rate, and the incidence of post-vaccine AEs.

Studies involving subjects who were children, pregnant women, received only one dose of the COVID-19 vaccine or lacked sufficient data were excluded, along with non-English publications, and those that replicated a prior study.

### 2.3 Study selection and data extraction

Studies were screened by two independent reviewers (Hui-Jun Li and Yang-Yang Yao). A third reviewer (Chao Zhang) was consulted when there was uncertainty about including a study. The following data were extracted from studies that met the inclusion criteria: name of the first author, year of publication, subject demographic information (mean age and gender), type of vaccine administered, dose of vaccine administered, time between vaccine dosages, the time between vaccination and serologic diagnosis, the number of occurrences of humoral immune responses and antibody levels, the number of occurrences of cellular immune responses, the number of post-vaccine AEs, and the number of people with post-vaccine AEs. Studies with multiple time points for serological diagnosis or more than one type of vulnerable population were defined as multiple studies during data extraction. If the continuous variable was shown as the median and interquartile range (IQR), data were extracted by estimating the sample mean and standard deviation based on the approximate formula for optimal weights (Shi et al., 2020).

### 2.4 Quality assessment

Two reviewers (Hui-Jun Li and Cheng-Yang Huang) independently used the Cochrane Risk of Bias 2.0 (RoB-2.0) to assess the quality of the included literature. The selected items included confounding bias, subject selection bias, intervention classification bias, bias in deviation from established interventions, missing data bias, endpoint measurement bias, and selective reporting bias. Responses to each question were selected from “Yes,” “Probably Yes,” “No,” “Probably No,” “No Information” and “Not Applicable.” Any disagreement between the two reviewers was resolved by a third reviewer (Shao-Juan Chen).

### 2.5 Statistical analysis

The effect size for outcomes that belonged to dichotomous data was presented as the odds ratio (OR) calculated using the Mantel-Haenszel method, with a corresponding 95% confidence interval (CI). For continuous data on outcomes, the effect size of the standard mean difference (SMD) was used with a 95% CI. Heterogeneity was assessed by calculating the I^2^ and *p* values. Data were analyzed using a random-effects model When *p* < 0.1 and I^2^ >40%, the data were analyzed using a random-effects model, and when *p* > 0.1 and I^2^ <40%, the data were analyzed using a fixed-effects model. According to Cochrane’s handbook, an I^2^ value ≤ 40% represents mild heterogeneity and a value > 40% represents significant heterogeneity. To further examine heterogeneity, various confounding factors were introduced into the subgroup analysis. IgG antibody levels were analysed in subgroups by type of disease [hemodialysis, organ transplant (kidney or liver), solid cancer, hematological malignancies (multiple myeloma, myeloproliferative neoplasms, and other haematological cancers)], elderly (yes/no), vaccination subtype (BNT162b2 or BNT162b2 mixed with other vaccines), vaccination dose (first and second), the time between doses (≤21 days or >21 days), number of days from vaccination to COVID-19 serologic test (≤21 days after the first vaccination dose, >21 days after the first vaccination dose, ≤21 days after the second vaccination dose, and >21 days after the second vaccination dose), antibody detection method [enzyme-linked immunosorbent assay (ELISA) and LIAISON], type of antibodies (anti-spike, anti-S1, anti-N, anti-S, and anti-RBD) and COVID-19 infection status (absence of infection, infection after vaccination, uncertain about infection). RevMan 5.4 was used for all statistical analyses.

## 3 Results

### 3.1 Study selection

**FIGURE 1:**
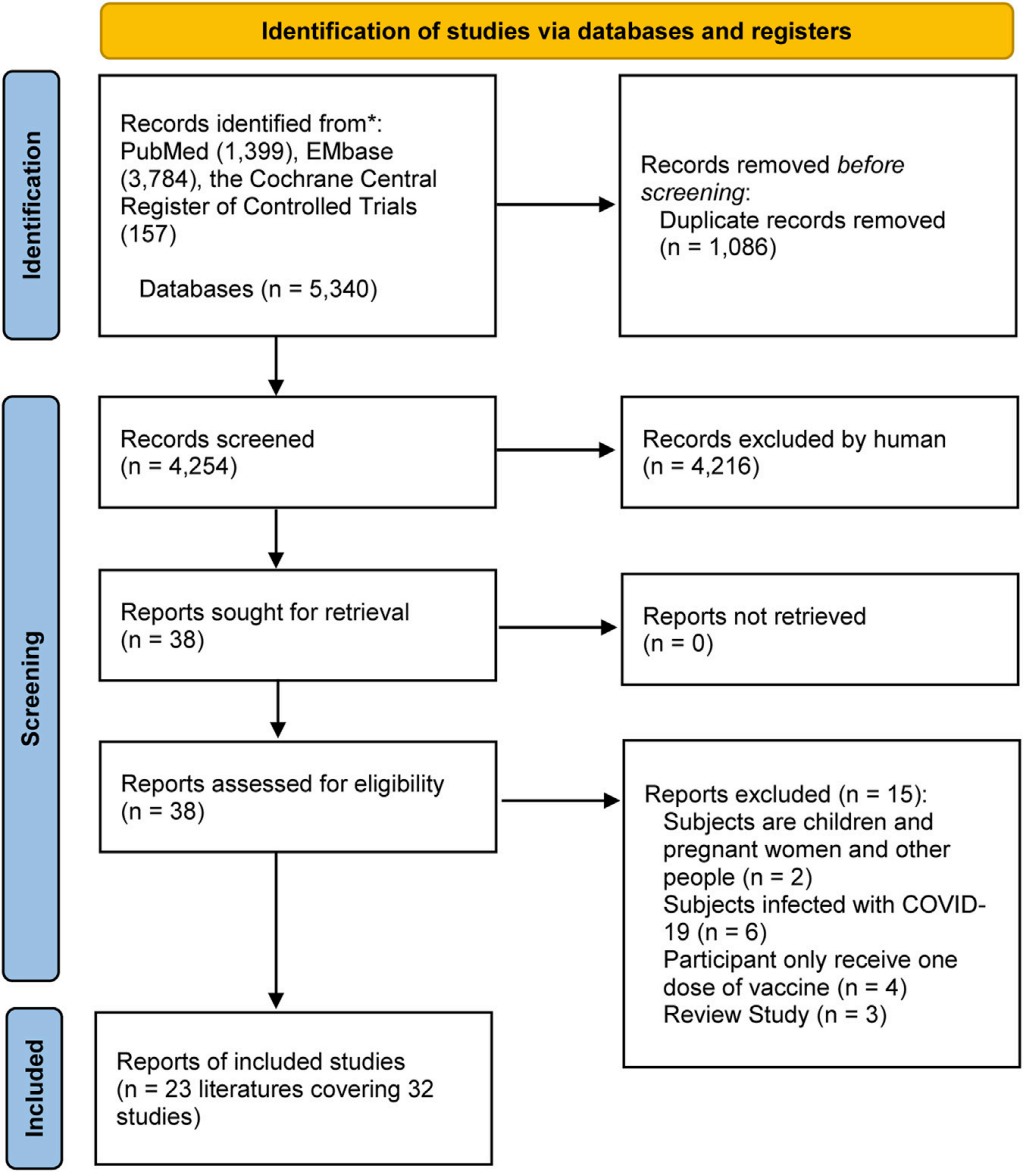
Literature screening for inclusion of studies.

A total of 5,340 articles were selected for the initial screening and after reading the titles and abstracts, 5,317 that did not meet the inclusion criteria were excluded. A total of 23 articles (Grupper et al., 2021a; Palich et al., 2021a; Grupper et al., 2021b; Palich et al., 2021b; Geisen et al., 2021; Goupil et al., 2021; Herzog Tzarfati et al., 2021; Korth et al., 2021; Levy et al., 2021; Massarweh et al., 2021; Monin et al., 2021; Moor et al., 2021; Pimpinelli et al., 2021; Rabinowich et al., 2021; Sattler et al., 2021; Speer et al., 2021; Stumpf et al., 2021; Tzioufas et al., 2021; Waissengrin et al., 2021; Waldhorn et al., 2021; Yanay et al., 2021; Ligumsky et al., 2022; Shem-Tov et al., 2022) covering 32 observational studies were finally included. The specific screening process is shown in Figure 1.

### 3.2 Study characteristics

The characteristics of the 32 observational studies (Grupper et al., 2021a; Palich et al., 2021a; Grupper et al., 2021b; Palich et al., 2021b; Geisen et al., 2021; Goupil et al., 2021; Herzog Tzarfati et al., 2021; Korth et al., 2021; Levy et al., 2021; Massarweh et al., 2021; Monin et al., 2021; Moor et al., 2021; Pimpinelli et al., 2021; Rabinowich et al., 2021; Sattler et al., 2021; Speer et al., 2021; Stumpf et al., 2021; Tzioufas et al., 2021; Waissengrin et al., 2021; Waldhorn et al., 2021; Yanay et al., 2021; Ligumsky et al., 2022; Shem-Tov et al., 2022), which included 4,875 vulnerable subjects and 2,285 healthy subjects, are shown in Table 1. The study subjects included cancer patients receiving immune checkpoint inhibitors, immunosuppressive therapy for chronic inflammatory diseases, maintenance hemodialysis, kidney and liver transplants, as well as those with solid cancers, hematologic malignancies, a history of CD20 B-cell depletion therapy, myeloproliferative tumors, human immunodeficiency virus (HIV) infection, autoimmune rheumatism, recipients of hematopoietic stem cell transplantation, and systemic autoimmune diseases. The subjects in this study were all ≥18 years of age and none had been infected with COVID-19 prior to vaccination. Most subjects received the BNT162b2 vaccination. The interval between the first and second vaccine dose was 3 weeks in most cases, and the interval between vaccination and serological diagnosis was 1–8 weeks. Of the 23 included studies, one (Speer et al., 2021) explicitly excluded those infected with COVID-19 after vaccination, seven (Palich et al., 2021a; Massarweh et al., 2021; Rabinowich et al., 2021; Sattler et al., 2021; Stumpf et al., 2021; Tzioufas et al., 2021; Shem-Tov et al., 2022) used nucleic acid testing to ensure that the included subjects were not infected with COVID-19 prior to vaccination, five (Grupper et al., 2021b; Goupil et al., 2021; Monin et al., 2021; Yanay et al., 2021; Ligumsky et al., 2022) documented subjects with asymptomatic or symptomatic infection after vaccination, and nine (Grupper et al., 2021a; Palich et al., 2021b; Geisen et al., 2021; Korth et al., 2021; Levy et al., 2021; Moor et al., 2021; Tzioufas et al., 2021; Waissengrin et al., 2021; Waldhorn et al., 2021) included a statement that the included subjects did not have COVID-19 prior to vaccination. Most studies measured the post-vaccine humoral immune response using an enzyme-linked immunosorbent assay (ELISA) or LIAISON. Individual studies assessed cellular immune responses using an interferon- γ release assay (IGRA) or fluorescent spot analysis of interferon-gamma (IFN-γ)-producing and interleukin-2 (IL-2)-producing SARS-CoV-2 reactive T cells.

**TABLE 1:** Characteristics of included observational studies.

Author	Year	Vulnerable populations	Sample	Female	Vaccination	Design of serologic test	Testing time
Geisen	2021	Immunosuppressive therapies for CIDS	26/42	46	mRNA vaccines from either BioNtech/Pfizer or Mode RNA	ELISA (anti-spike antibodies)	7 days after the second dosage
Goupil	2021	Hemodialysis	131/20	57	One dosage of BNT162b2	ELISA (anti-RBD antibodies)	4 weeks after vaccination; 8 weeks after vaccination
Grupper	2021	Maintenance hemodialysis	56/95	83	Two dosages of the BNT162b2 (pfizer-biontech) vaccine	the Abbott SARS-CoV-2 IgG chemiluminescent immunoassay (anti-S1)	1 week after vaccination
Grupper	2021	Kidney transplant	136/25	42	Two dosages of BNT162b2	LIAISON SARS-CoV-2 S1/S2 IgG chemiluminescent assay	10–20 days after the second vaccine dose
Korth	2021	Kidney transplant	23/23	26	Two dosages of BNT162b2	anti-SARS-CoV-2 IgG CLIA (anti-spike antibodies)	14 days after the second vaccination
Levy	2021	HIV-1	143/261	207	Two dosages of BNT162b2	ELISA anti-RBD	18/26 days
Ligumsky	2021	Solid cancer	326/164	303	Two dosages of BNT162b2	anti-S IgG assay chemiluminescent microparticle immunoassay	78 days
Massarweh	2021	Solid cancer	102/78	97	Two dosages of BNT162b2	chemiluminescent immunoassay (anti-spike antibodies)	38 days after the second vaccine dosage in the patient group; 40 days after the second vaccine dosage in the controls
Monin	2021	Solid cancer	56/34	NA	Two dosages of the BNT162b2 (pfizer-biontech) vaccine	ELISA (anti-spike antibodies)	3 and 5 weeks after the first vaccine dosage
Monin	2021	Hematological cancer	44/34	NA	Two dosages of the BNT162b2 (pfizer-biontech) vaccine	ELISA (anti-spike antibodies)	3 and 5 weeks after the first vaccine dosage
Moor	2021	CD20 B-cell depleting therapy	96/29	70	Two dosages of either the pfizer–biontech BNT162b2 vaccine or the mode RNA mRNA-1273 vaccine	ELISA (anti-spike antibodies)	4 weeks after the twice vaccine dosage
Palich	2021	Solid cancer	110/25	84	One dosage of BNT162b2	The Abbott SARS-CoV-2 IgG chemiluminescent immunoassay (anti-S) (anti-N)	21 days after the first vaccine dosage
Palich	2021	Solid cancer	223/49	174	Two dosages of BNT162b2	anti-S IgG CMIA, anti-S IgG ECLIA	3–4 weeks after the second vaccination
Pimpinelli	2021	Multiple myeloma	42/36	37	One dosage of BNT162b2	The LIAISON SARS-CoV-2 S1/S2 IgG test	3 weeks after the first vaccine dosage
Pimpinelli	2021	Myeloproliferative neoplasms	50/36	42	One dosage of BNT162b2	The LIAISON SARS-CoV-2 S1/S2 IgG test	3 weeks after the first vaccine dosage
Pimpinelli	2021	Multiple myeloma	42/36	37	Two dosages of BNT162b2	The LIAISON SARS-CoV-2 S1/S2 IgG test	3 weeks after the twice vaccine dosage
Pimpinelli	2021	Myeloproliferative neoplasms	50/36	42	Two dosages of BNT162b2	The LIAISON SARS-CoV-2 S1/S2 IgG test	3 weeks after the twice vaccine dosage
Rabinowich	2021	Liver transplant recipients	80/25	41	Sars-cov-2 vaccination	LIAISON SARS-CoV-2 S1/S2 IgG chemiluminescent assay	10–20 days after the second vaccine dosage
Sattler	2021	Kidney transplant	39/39	30	Two dosages of BNT162b2	ELISA (anti-S1)	8 ± 1 day after the second vaccine
Sattler	2021	Hemodialysis	26/39	28	Two dosages of BNT162b2	ELISA (anti-S1)	8 ± 1 day after the second vaccine
Shem-Tov	2021	Hematopoietic stem cell transplantation recipients	152/272	262	Two dosages of BNT162b2	ELISA anti-RBD	2–4 weeks/28 days after the second vaccination
Speer	2021	Hemodialysis	22/46	37	One dosage of BNT162b2	ELISA (anti-S1)	18/19 days after one dosage
Speer	2021	Hemodialysis	17/46	NA	Two dosages of BNT162b2	ELISA (anti-S1)	20/20 days after two dosages
Stumpf	2021	Dialysis patients	1256/144	548	One dosage of either BNT162b2 vaccine or the mRNA-1273 vaccine	ELISA (anti-S1)	3–4 weeks after first vaccination
Stumpf	2021	Dialysis patients	1256/144	548	Two dosages of either BNT162b2 vaccine or the mRNA-1273 vaccine	ELISA (anti-S1)	8 weeks after first vaccination
Stumpf	2021	Kidney transplant recipient	368/144	237	One dosages of either the BNT162b2 vaccine or the mRNA-1273 vaccine	ELISA (anti-S1)	3–4 weeks after first vaccination
Stumpf	2021	Kidney transplant recipient	368/144	237	Two dosages of either the BNT162b2 vaccine or the mRNA-1273 vaccine	ELISA (anti-S1)	8 weeks after first vaccination
Tzarfati	2021	Hematological cancer	315/108	200	Two dosages of BNT162b2	Chemiluminescence immunoassay (anti-S1/S2)	1–2 weeks after the second vaccination
Tzioufas	2021	Systemic autoimmune and autoinflammatory rheumatic diseases	605/116	501	Two dosages of BNT162b2 or the mRNA-1273	ELISA anti-S1	4 weeks after vaccination
Waissengrin	2021	Cancer treated with immune checkpoint inhibitors	134/134	NA	The pfizer BNT162b2 mRNA vaccine	NA	19 days after the second vaccine dosage
Waldhorn	2021	Solid cancer	154/135	145	Two dosages of BNT162b2	LIAISON SARS-CoV-2 S1/S2 IgG	2 weeks after vaccination
Yanay	2021	Chronic dialysis	160/132	124	Two dosages of BNT162b2	LIAISON SARS-CoV-2 S1/S2 IgG	21–35 days after the second dosage of the vaccine

Note: CIDs, Chronic inflammatory diseases; HIV-1, Human immunodeficiency virus 1; ELISA, Enzyme linked immunosorbent assay; NA, not applicable.

### 3.3 Quality evaluation

The results of a qualitative evaluation of this study are shown in Table 2. None of the 24 studies had a potential confounding effect on the intervention. The selection of study subjects was not based on individual characteristics observed after the start of the intervention, and the intervention and follow-up did not coincide for most subjects. While the intervention groups were clearly defined, information used to characterize the intervention groups was not recorded at the start of the intervention, and the delineation of the intervention groups was not affected by knowledge or risks associated with the outcome. Significant concomitant interventions were likely balanced between intervention groups, and most subjects successfully implemented and were compliant with the assigned interventions. Knowledge about the intervention did not affect the outcome measurements, the outcome assessor was aware of the intervention the subject received, and there was no systematic error in the outcome measure associated with receiving the intervention. Outcomes were available for all subjects in all studies, and there was no selective reporting of multiple measures for specific outcomes or the effects of multiple analyses of the intervention-outcome relationship.

**TABLE 2:** Evaluation of the quality of included studies.

Evaluation field	Confounding bias	Subject selection bias			Intervention classification bias			Bias in deviation from established interventions			Missing data bias					Endpoint measurement bias				Selective reporting bias	
	①	②	③	④	⑤	⑥	⑦	⑧	⑨	⑩	⑪	⑫	⑬	⑭	⑮	⑯	⑰	⑱	⑲	⑳	㉑
Geisen, 2021	N	N	N	NA	Y	N	N	PY	Y	Y	Y	NA	NA	NA	NA	PN	Y	NA	N	N	N
Goupil, 2021	N	N	N	NA	Y	N	N	PY	Y	Y	Y	NA	NA	NA	NA	PN	Y	NA	N	N	N
Grupper, 2021	N	N	N	NA	Y	N	N	PY	Y	Y	Y	NA	NA	NA	NA	PN	Y	NA	N	N	N
Grupper, 2021	N	N	N	NA	Y	N	N	PY	Y	Y	Y	NA	NA	NA	NA	PN	Y	NA	N	N	N
Korth, 2021	N	N	N	NA	Y	N	N	PY	Y	Y	Y	NA	NA	NA	NA	PN	Y	NA	N	N	N
Levy, 2021	N	N	N	NA	Y	N	N	PY	Y	Y	Y	NA	NA	NA	NA	PN	Y	NA	N	N	N
Ligumsky, 2021	N	N	N	NA	Y	N	N	PY	Y	Y	Y	NA	NA	NA	NA	PN	Y	NA	N	N	N
Massarweh, 2021	N	N	N	NA	Y	N	N	PY	Y	Y	Y	NA	NA	NA	NA	PN	Y	NA	N	N	N
Monin, 2021	N	N	N	NA	Y	N	N	PY	Y	Y	Y	NA	NA	NA	NA	PN	Y	NA	N	N	N
Moor, 2021	N	N	N	NA	Y	N	N	PY	Y	Y	Y	NA	NA	NA	NA	PN	Y	NA	N	N	N
Palich, 2021	N	N	N	NA	Y	N	N	PY	Y	Y	Y	NA	NA	NA	NA	PN	Y	NA	N	N	N
Palich, 2021	N	N	N	NA	Y	N	N	PY	Y	Y	Y	NA	NA	NA	NA	PN	Y	NA	N	N	N
Pimpinelli, 2021	N	N	N	NA	Y	N	N	PY	Y	Y	Y	NA	NA	NA	NA	PN	Y	NA	N	N	N
Rabinowich, 2021	N	N	N	NA	Y	N	N	PY	Y	Y	Y	NA	NA	NA	NA	PN	Y	NA	N	N	N
Sattler, 2021	N	N	N	NA	Y	N	N	PY	Y	Y	Y	NA	NA	NA	NA	PN	Y	NA	N	N	N
Shem-Tov, 2021	N	N	N	NA	Y	N	N	PY	Y	Y	Y	NA	NA	NA	NA	PN	Y	NA	N	N	N
Speer, 2021	N	N	N	NA	Y	N	N	PY	Y	Y	Y	NA	NA	NA	NA	PN	Y	NA	N	N	N
Stumpf, 2021	N	N	N	NA	Y	N	N	PY	Y	Y	Y	NA	NA	NA	NA	PN	Y	NA	N	N	N
Tzarfati, 2021	N	N	N	NA	Y	N	N	PY	Y	Y	Y	NA	NA	NA	NA	PN	Y	NA	N	N	N
Tzioufas, 2021	N	N	N	NA	Y	N	N	PY	Y	Y	Y	NA	NA	NA	NA	PN	Y	NA	N	N	N
Waissengrin, 2021	N	N	N	NA	Y	N	N	PY	Y	Y	Y	NA	NA	NA	NA	PN	Y	NA	N	N	N
Waldhorn, 2021	N	N	N	NA	Y	N	N	PY	Y	Y	Y	NA	NA	NA	NA	PN	Y	NA	N	N	N
Yanay, 2021	N	N	N	NA	Y	N	N	PY	Y	Y	Y	NA	NA	NA	NA	PN	Y	NA	N	N	N

**Note:** Y, yes; PY, probably yes; N, no; PN, probably no; NI, no information; NA, not applicable; ①: Whether there are potential confounding factors in the study on the effect of the intervention; ②: Whether the selection of study subjects (for inclusion in the study or analysis) was based on individual characteristics observed after the start of the intervention; ③: Whether the stem of most subjects is consistent with the start of the follow-up pre; ④: Whether the correction technique applied corrects for selectivity bias; ⑤: Whether the intervention group was clearly defined; ⑥: Whether defining information about the intervention group is recorded at the start of the intervention; ⑦: Whether the delineation of the intervention group is influenced by knowledge of the outcome or the risk associated with the outcome; ⑧: Balance of important concomitant interventions between intervention groups; ⑨: Whether the intervention was successfully implemented for the majority of subjects; ⑩: Whether study subjects comply with the assigned intervention; ⑪: Availability of outcome data for all or nearly all subjects; ⑫: Whether subjects were excluded due to missing data on intervention status; ⑬: Whether subjects were excluded due to missing data for other variables required for the analysis; ⑭: Whether the proportion of missing data and the reasons for missing data are similar between groups; ⑮: Whether evidence exists that the results are still robust after data loss; ⑯: Knowledge about the intervention, whether it affects outcome measures; ⑰: Whether the outcome assessor was aware of the intervention the subject received; ⑱: Whether the outcome assessment methods were comparable between intervention groups; ⑲: Presence of systematic errors associated with the accepted intervention at the time of the ending measurement; ⑳: Whether to selectively report multiple outcome measures for specific outcome domains; ㉑: Whether to selectively report effect values for multiple analysis modalities for the intervention-outcome relationship.

### 3.4 Humoral immune responses

#### 3.4.1 IgG

**FIGURE 2:**
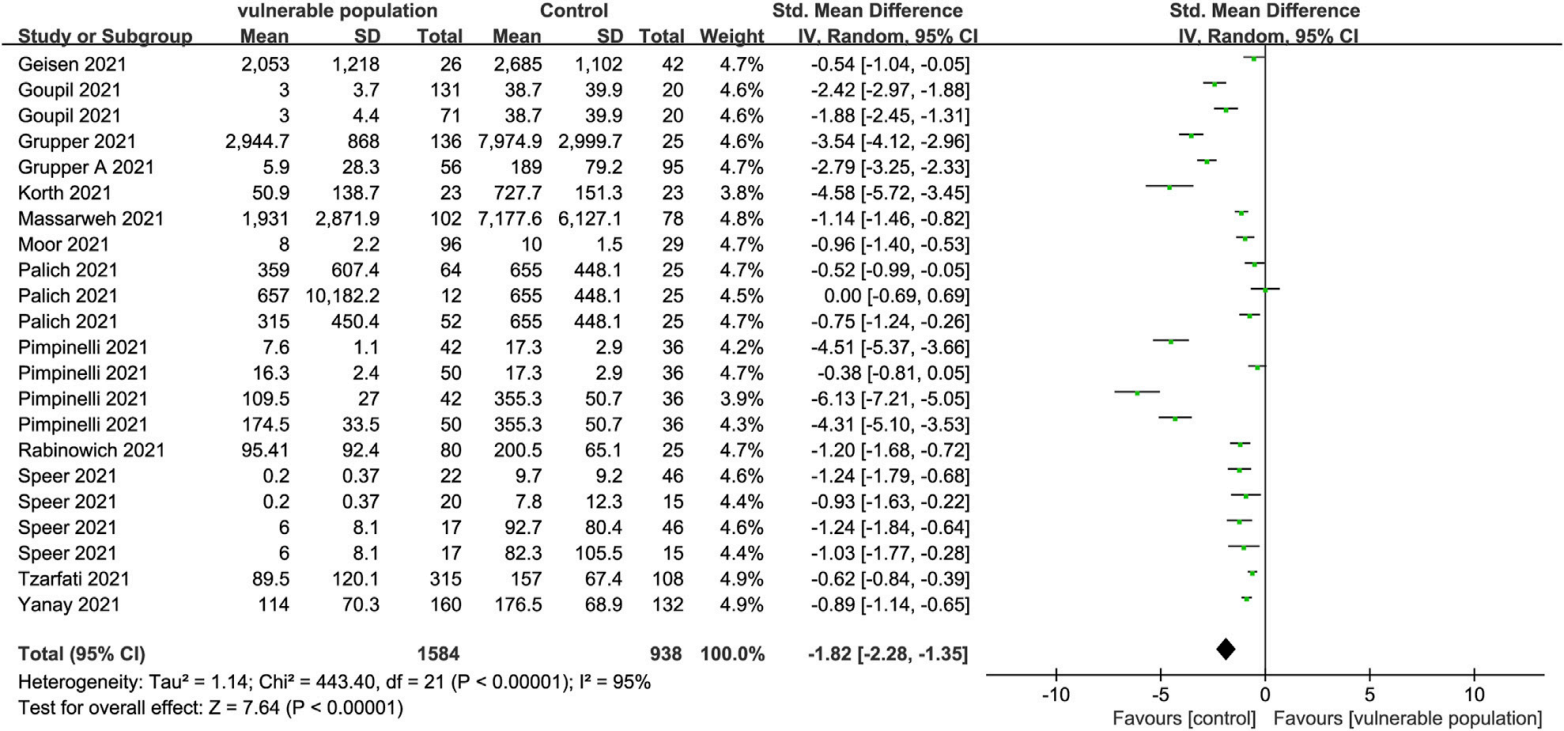
Forest plot showing the levels of IgG antibodies in sera of vulnerable and healthy populations.

A total of 13 articles describing 22 studies (Grupper et al., 2021a; Palich et al., 2021a; Grupper et al., 2021b; Geisen et al., 2021; Goupil et al., 2021; Herzog Tzarfati et al., 2021; Korth et al., 2021; Massarweh et al., 2021; Moor et al., 2021; Pimpinelli et al., 2021; Rabinowich et al., 2021; Speer et al., 2021; Yanay et al., 2021) that included 1,303 vulnerable subjects and 720 healthy subjects reported serum anti-SARS-CoV-2 IgG antibody levels. The enrolled vulnerable populations included patients receiving immunosuppressive therapy for chronic inflammatory diseases, hemodialysis, and kidney or liver transplants, as well as those with solid cancers, a history of CD20 B-cell depletion therapy, and hematologic malignancy. Anti-spiking IgG antibodies were significantly lower in the vulnerable than in the healthy populations (SMD = −1.82, 95% CI [−2.28, −1.35]) (Figure 2).

**FIGURE 3:**
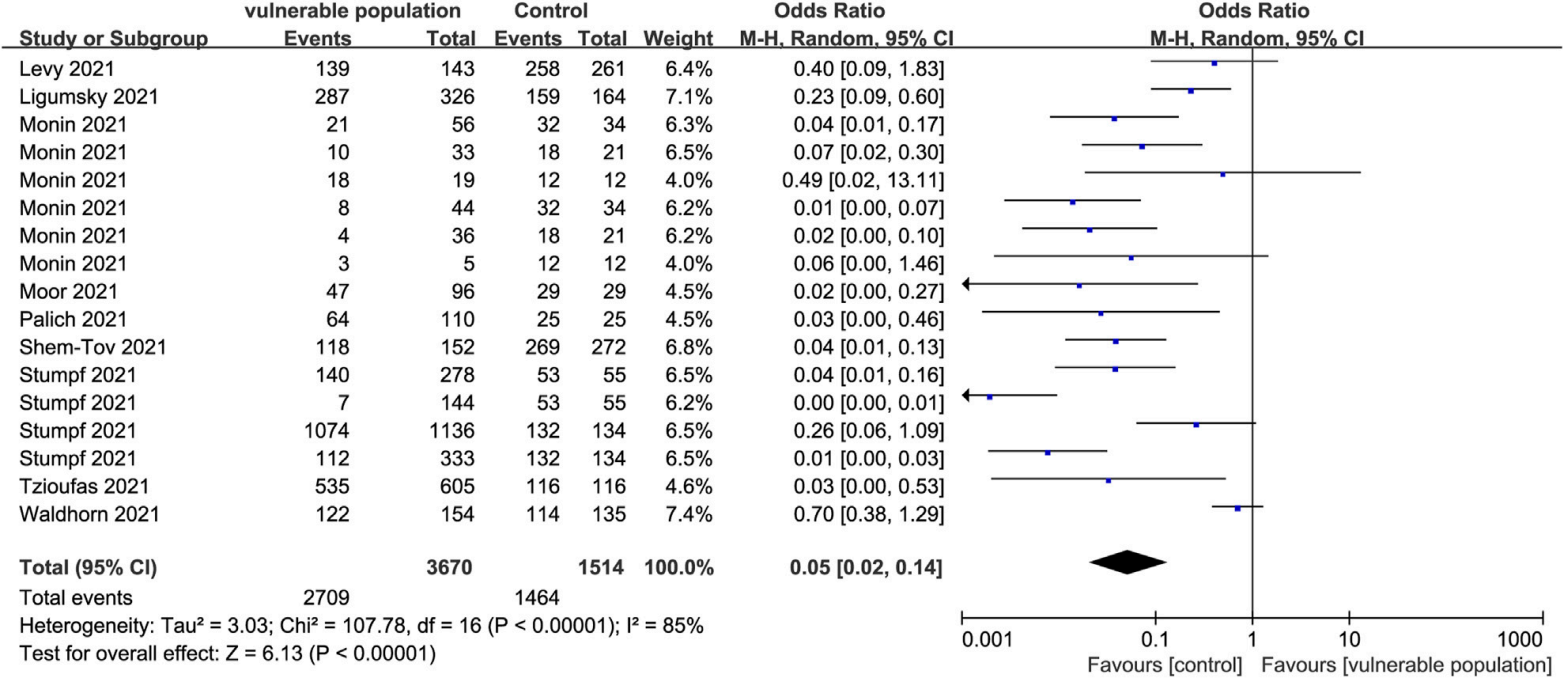
Forest plot showing the detection rate of positive IgG antibodies in sera of vulnerable and healthy populations.

A total of 17 studies (Palich et al., 2021a; Levy et al., 2021; Monin et al., 2021; Moor et al., 2021; Stumpf et al., 2021; Tzioufas et al., 2021; Waldhorn et al., 2021; Ligumsky et al., 2022; Shem-Tov et al., 2022) involving 3,100 vulnerable subjects and 1,259 healthy participants reported the proportion of individuals with positive IgG antibody test results. The vulnerable subjects could be classified into eight groups including patients with solid cancers, hematological illnesses, renal transplant, a history of CD20 B-cell depletion therapy, HIV, autoinflammatory rheumatic diseases, hematopoietic stem cell transplantation, and systemic autoimmune diseases. A positive antibody reaction was reported as follows: serum samples at a concentration of 50% of maximal effect (EC50) value using GraphPad Prism at a 1:25 dilution or an optical density (OD) ≥25 at 405 nm measured after four further dilutions (Levy et al., 2021); samples with an OD ratio >1 at 450–620 nm to the OD of the calibrator (Ligumsky et al., 2022); an anti-N IgG assay value ≥ 0.8 UA/mL and an anti-s IgG assay value ≥ 50 UA/mL (Monin et al., 2021); new antibodies at the T1 or T2 phase (seroconversion) (Stumpf et al., 2021). Serological diagnostic time points were set selectively at 1–8 weeks post-vaccination in each study according to their respective conditions. Anti-S IgG antibody levels were lower in the vulnerable than in the healthy populations (OR = 0.05, 95% CI [0.02, 0.14]) (Figure 3).

#### 3.4.2 IgA

**FIGURE 4:**
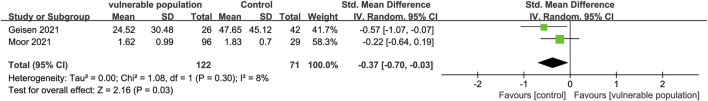
Forest plot showing the levels of IgA antibodies in sera of vulnerable and healthy populations.

Two studies (Geisen et al., 2021; Moor et al., 2021) examined IgA antibody levels in the serum of 122 vulnerable patients (those receiving immunosuppressive therapy for chronic inflammatory diseases and those with a history of CD20 b-cell depletion therapy) and 71 healthy subjects. Vulnerable subjects had significantly lower IgA antibody levels than healthy subjects (SMD = −0.37, 95% CI [−0.70, −0.03]) (Figure 4).

**FIGURE 5:**
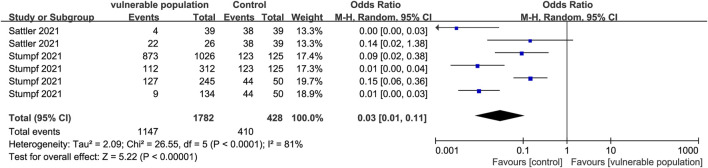
Forest plot showing the positive detection rate of IgG antibodies in sera of vulnerable and healthy populations.

Two studies (Sattler et al., 2021; Stumpf et al., 2021) reported the number of subjects with positive IgA antibody responses, determined by OD ratios ≥1.1, and the presence of new antibodies at T1 or T2 (seroconversion). They included 1,403 subjects from vulnerable populations (hemodialysis and renal transplant recipients) and 328 subjects from healthy populations. The proportion of IgA-positive subjects was lower in the vulnerable than in the healthy groups (OR = 0.03, 95% CI [0.01, 0.11]) (Figure 5).

#### 3.4.3 IgM

**FIGURE 6:**
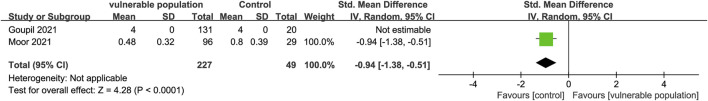
Forest plot showing the levels of IgM antibodies in the sera of vulnerable and healthy populations.

Two studies (Goupil et al., 2021; Moor et al., 2021) enrolling 227 vulnerable subjects (hemodialysis patients and those with a history of CD20 b-cell depletion therapy) and 49 healthy subjects reported serum IgM antibody levels. The vulnerable subjects had significantly lower levels of IgM antibodies than the healthy subjects (SMD = −0.94, 95% CI [−1.38, −0.51]) (Figure 6).

#### 3.4.4 Neutralizing antibody

**FIGURE 7:**
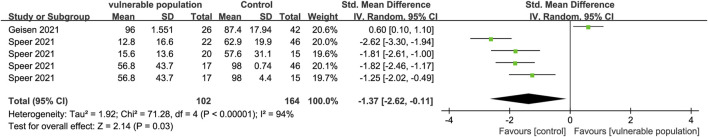
Forest plot showing the levels of neutralizing antibodies in sera of vulnerable and healthy populations.

Two studies (Geisen et al., 2021; Speer et al., 2021) including 48 vulnerable individuals (those receiving hemodialysis and those with chronic inflammatory diseases) and 88 healthy subjects, reported serum levels of neutralizing antibodies. Antibody levels were lower in the vulnerable groups than in the healthy groups (SMD = −1.37, 95% CI [−2.62, −0.11]) (Figure 7). One study (Grupper et al., 2021a) reported that kidney transplant recipients had relatively lower levels of serum antibodies than healthy subjects.

### 3.5 Cellular immune responses

**FIGURE 8:**
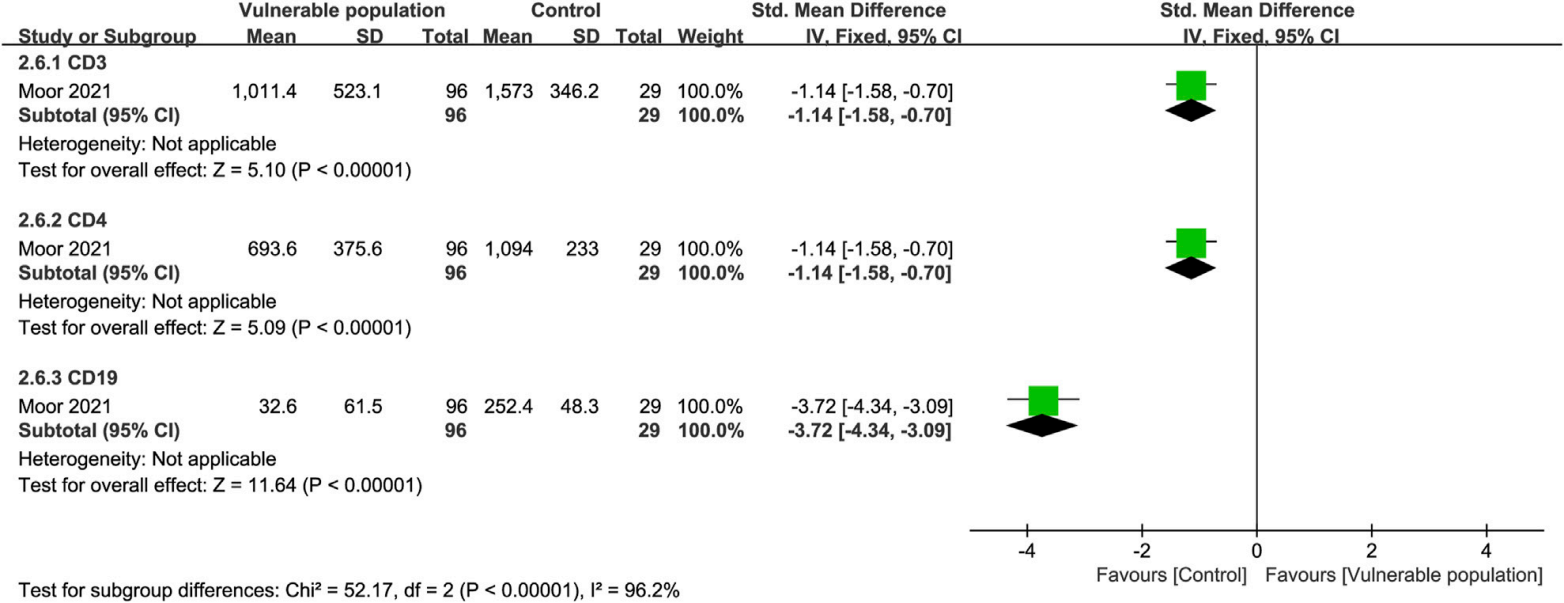
Forest plot showing the levels of CD3, CD4 and CD19 cells in the sera of vulnerable and healthy populations.

One study (Moor et al., 2021) examined CD3, CD4, and CD19 expression in the plasma of patients with a history of CD20 b-cell depletion therapy and healthy individuals. There were fewer CD3 (SMD = −1.14, 95% CI [−1.58, −0.70]), CD4 (SMD = −1.14, 95% CI [−1.58, −0.70]), and CD19 (SMD = −3.72, 95% CI [−4.34, −3.09]) expressing cells in the plasma of the vulnerable patients than the healthy subjects (Figure 8).

**FIGURE 9:**
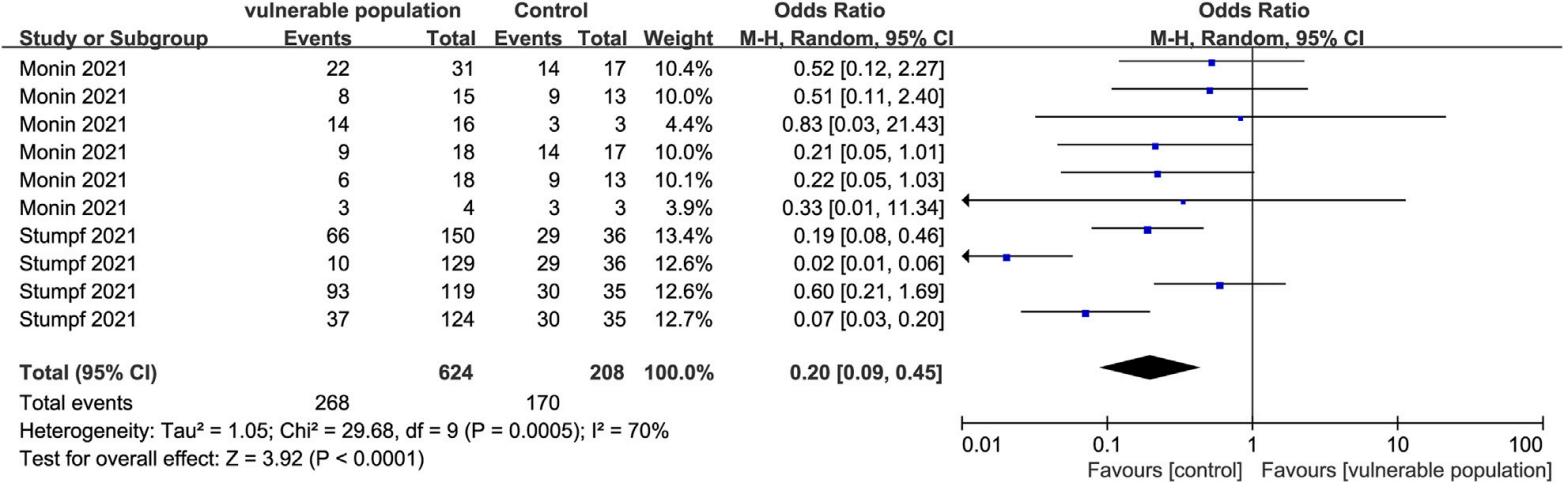
Forest plot showing the prevalence of positive cellular immune responses in vulnerable and healthy populations.

Two studies (Monin et al., 2021; Stumpf et al., 2021) enrolling 328 vulnerable people (hemodialysis patients and renal transplant recipients) and 106 healthy people, reported the number of subjects who developed a cellular immune response. The criteria for determining a positive antibody reaction were reported as follows: an EC50 that did not reach 1:25; an OD at 405 nm that was 4-fold above background and assigned a value of 25; IFN-γ release ≥100 MIU/mL. Vulnerable subjects had lower cellular immune responses than healthy subjects (OR = 0.20, 95% CI [0.09, 0.45]) (Figure 9).

### 3.6 Adverse events

#### 3.6.1 Fever

Three studies (Geisen et al., 2021; Pimpinelli et al., 2021; Waissengrin et al., 2021), including 255 vulnerable subjects and 215 healthy subjects, reported fever as an AE. Vulnerable populations included those receiving immunosuppressive treatment for chronic inflammatory diseases, hematologic malignancy patients, and cancer patients receiving immune checkpoint inhibitors. There was no statistical difference between the vulnerable and healthy groups (OR = 2.53, 95% CI [0.11, 60.86]).

#### 3.6.2 Chills

Three trials (Geisen et al., 2021; Pimpinelli et al., 2021; Waissengrin et al., 2021) including 255 vulnerable subjects and 215 healthy subjects reported chills as AEs. Vulnerable subjects included patients receiving immunosuppressive therapies for chronic inflammatory diseases, hematologic malignancy patients, and cancer patients receiving immune checkpoint inhibitors. There was no statistical difference between the vulnerable and healthy groups (OR = 2.03, 95% CI [0.08, 53.85]).

#### 3.6.3 Myalgia

AEs of myalgia occurred in four trials (Geisen et al., 2021; Pimpinelli et al., 2021; Waissengrin et al., 2021; Ligumsky et al., 2022) including 581 vulnerable subjects and 379 healthy subjects. Enrolled vulnerable subjects included patients receiving immunosuppressive therapies for chronic inflammatory diseases, hematologic malignancy patients, cancer patients receiving immune checkpoint inhibitors, and solid cancer patients. There was no statistical difference between the vulnerable and healthy groups (OR = 10.31, 95% CI [0.56, 191.08]).

#### 3.6.4 Local pain at the site of injection

AEs of local pain at the injection site were reported by three studies (Grupper et al., 2021a; Geisen et al., 2021; Ligumsky et al., 2022) including 488 vulnerable subjects and 231 healthy subjects. Vulnerable populations included patients who received immunosuppressive therapy for chronic inflammatory diseases, renal transplant recipients, and solid cancer patients. There was no statistical difference between the vulnerable and healthy groups (OR = 17.83, 95% CI [0.32, 989.06]).

#### 3.6.5 Headache

Headache AEs occurred in four studies (Geisen et al., 2021; Pimpinelli et al., 2021; Waissengrin et al., 2021; Ligumsky et al., 2022) of 673 vulnerable subjects and 415 healthy subjects. Vulnerable subjects included patients receiving immunosuppressive therapies for chronic inflammatory diseases, hematologic malignancy patients, solid cancer patients, and cancer patients receiving immune checkpoint inhibitors. Only one patient with multiple myeloma and myeloproliferative neoplasm experienced a headache during the first dose of BNT162b2, and two patients experienced a headache during the second dose of BNT162b2. There was no statistical difference between the vulnerable and healthy groups (OR = 4.80, 95% CI [0.75, 30.71]).

#### 3.6.6 Tenderness

Injection-related tenderness AEs were reported in one study (Pimpinelli et al., 2021), including 92 vulnerable and 36 healthy subjects. Vulnerable subjects included patients receiving immunosuppressive therapy for chronic inflammatory diseases and renal transplant recipients. There was no statistical difference between the vulnerable and healthy groups (OR = 7.30, 95% CI [0.95, 55.91]).

#### 3.6.7 Fatigue

AEs of fatigue were observed in three studies (Geisen et al., 2021; Waissengrin et al., 2021; Ligumsky et al., 2022) including 489 vulnerable and 343 healthy subjects. Vulnerable subjects included patients treated with immune checkpoint inhibitors, patients receiving immunosuppressive therapy for chronic inflammatory diseases, and solid cancer patients. There was no statistical difference between the vulnerable and healthy groups (OR = 22.89, 95% CI [0.45, 1164.22]).

#### 3.6.8 Other adverse events

One study (Waissengrin et al., 2021) reported flu-like symptoms in three cancer patients receiving immune checkpoint inhibitors. Five patients (Pimpinelli et al., 2021) with multiple myeloma and myeloproliferative neoplasms, who received a single dose of BNT162b2, developed malaise. According to one study (Geisen et al., 2021), four of 26 immunosuppressed patients with chronic inflammatory diseases had arthralgia, two had local erythema, four had local swelling, three had swollen lymph nodes, nine required NSAIDs, and five had other AEs (not specified in the original article). Meanwhile, six of 42 subjects in the healthy group had arthralgia, two had local erythema, four had local swelling, four had swollen lymph nodes, ten required NSAIDs, and seven had other AEs (also not specified in the original article).

### 3.7 Stratification analysis

#### 3.7.1 Vulnerable populations

Serum IgG antibody levels were lower in patients receiving hemodialysis (Grupper et al., 2021b; Goupil et al., 2021; Speer et al., 2021; Yanay et al., 2021) (SMD = −1.88, 95% CI [−3.03, −0.72]) or organ transplants (Grupper et al., 2021a; Korth et al., 2021; Rabinowich et al., 2021) (SMD = −2.78, 95% CI [−4.34, −1.22]) and those with hematological malignancies (Herzog Tzarfati et al., 2021; Pimpinelli et al., 2021) (SMD = −3.66, 95% CI [−7.15, −0.16]) or solid cancers (Palich et al., 2021b; Massarweh et al., 2021) (SMD = −0.85, 95% CI [−1.47, −0.24]) than in healthy individuals. Vulnerable patients with an average age >60 years were classified as elderly. A total of 1,116 vulnerable elderly subjects (Grupper et al., 2021b; Palich et al., 2021b; Goupil et al., 2021; Herzog Tzarfati et al., 2021; Massarweh et al., 2021; Moor et al., 2021; Pimpinelli et al., 2021; Rabinowich et al., 2021; Yanay et al., 2021) had lower IgG antibody levels than their healthy counterparts (SMD = −2.01, 95% CI [−2.68, −1.34]) (Table 3).

**TABLE 3:** Subgroup analysis of IgG antibody levels in sera from vulnerable and healthy populations.

Subgroup	Subgroup analysis	N	Sample	SMD [95%CI]	P for SMD	I^2^	P for I^2^
Types of vulnerable people	Hemodialysis	4	384/223	−1.88 [−3.03, −0.72]	0.001	96%	<0.00001
	Organ transplant	3	159/143	−2.78 [−4.34, −1.22]	0.0005	95%	<0.00001
	Solid cancer	2	166/103	−0.85 [−1.47, −0.24]	0.006	79%	0.03
	Hematological malignancies	3	407/180	−3.66 [−7.15, −0.16]	0.04	99%	<0.00001
	The elderly (>60 years)	10	1116/514	−2.01 [−2.68, −1.34]	<0.00001	96%	<0.00001
Types of vaccines	BNT162b2 only	12	1116/649	−2.30 [−2.99, −1.62]	<0.00001	97%	<0.00001
	BNT162b2 mixed with other vaccines	2	122/71	−0.77 [−1.18, −0.36]	<0.00001	36%	0.21
Injections for vaccines	One dosage	5	249/163	−1.66 [−2.79, −0.53]	0.004	95%	<0.00001
	Two dosages	12	1103/675	−2.22 [−2.89, −1.56]	<0.00001	97%	<0.00001
Interval time between vaccine dosages	≤ 21 days	4	284/192	−4.11 [−5.27, −2.95]	<0.00001	92%	<0.00001
	> 21 days	3	66/111	−2.04 [−3.82, −0.26]	0.02	95%	<0.00001
Days from vaccination dosage to COVID-19 Ab test	≤ 21 days after the first vaccination dosage	4	178/143	−1.62 [−3.00, −0.23]	0.02	96%	<0.00001
	> 21 days after the first vaccination dosage	1	71/20	−1.88 [−2.45, −1.31]	<0.00001	NA	NA
	≤ 21 days after the second vaccination dosages	9	745/436	−2.71 [−3.77, −1.65]	<0.00001	97%	<0.00001
	> 21 days after the second vaccination dosages	3	358/239	−0.98 [−1.16, −0.81]	<0.00001	0%	0.48
Antibody detection methods	ELISA	4	210/137	−1.14 [−1.67, −0.61]	<0.0001	76%	0.006
	LIAISON	10	1028/583	−2.47 [−3.25, −1.68]	<0.00001	97%	<0.00001
Types of antibodies	Anti-spike	11	1014/625	−2.01 [−2.66, −1.37]	<0.00001	96%	<0.00001
	Anti-S1	3	205/96	−1.84 [−3.53, −0.15]	0.03	95%	<0.00001
	Anti-RBD	1	71/20	−1.88 [−2.45, −1.31]	<0.00001	NA	NA
Whether subjects in the study were infected with COVID-19	Absence of infection	7	651/360	−1.31 [−1.90, −0.72]	<0.0001	93%	<0.00001
	Infection occurs after vaccination	3	287/284	−1.84 [−3.09, −0.60]	0.004	96%	<0.00001
	uncertainty about infection	3	255/77	−2.99 [−5.16, −0.82]	0.007	97%	<0.00001

Note: COVID-19 Ab, Coronavirus disease 2019 antibody; ELISA, Enzyme linked immunosorbent assay; SMD, standard mean difference; N, number of included studies; NA, Not applicable.

#### 3.7.2 Vaccination type

In the 12 studies (Grupper et al., 2021a; Grupper et al., 2021b; Palich et al., 2021b; Goupil et al., 2021; Herzog Tzarfati et al., 2021; Korth et al., 2021; Massarweh et al., 2021; Pimpinelli et al., 2021; Rabinowich et al., 2021; Speer et al., 2021; Yanay et al., 2021) that used the BNT162b2 vaccine alone, IgG antibody levels were lower in vulnerable than in healthy individuals (SMD = −2.30, 95% CI [−2.99, −1.62]). In the two studies (Geisen et al., 2021; Moor et al., 2021) in which subjects, including 122 vulnerable and 71 healthy individuals, received either the BNT162b2 or the Moderna mRNA-1273 vaccine, IgG antibody levels were also lower in vulnerable than healthy individuals (SMD = −0.77, 95% CI [−1.18, −0.36]) (Table 3).

#### 3.7.3 Vaccine doses

In five studies (Palich et al., 2021b; Goupil et al., 2021; Pimpinelli et al., 2021; Speer et al., 2021), including 249 vulnerable and 163 healthy subjects, in which only one vaccine dose was administered, the vulnerable subjects had weaker IgG antibody levels than the healthy individuals (SMD = −1.66, 95% CI [−2.79, −0.53]). In 12 studies (Grupper et al., 2021a; Grupper et al., 2021b; Geisen et al., 2021; Korth et al., 2021; Massarweh et al., 2021; Moor et al., 2021; Pimpinelli et al., 2021; Rabinowich et al., 2021; Speer et al., 2021; Tzioufas et al., 2021; Yanay et al., 2021) in which two vaccine doses were administered, IgG antibody levels were also lower in the vulnerable than in the healthy population (SMD = −2.22, 95% CI [−2.89, −1.56]) (Table 3).

#### 3.7.4 Interval time between vaccine doses

Of the studies in which two vaccine doses were administered, four studies (Grupper et al., 2021a; Grupper et al., 2021b; Pimpinelli et al., 2021), including 284 vulnerable and 192 healthy subjects, had an interval of ≤21 days between the two doses. IgG antibody levels were lower in the vulnerable than in the healthy subjects (SMD = −4.11, 95% CI [−5.27, −2.95]). Only three studies (Geisen et al., 2021; Korth et al., 2021; Speer et al., 2021) had an interval of >21 days between the two doses and the IgG antibody levels were lower in the vulnerable than in the healthy group (SMD = −2.04, 95% CI [−3.82, −0.26]) (Table 3).

#### 3.7.5 Time from vaccination to COVID-19 Ab testing

IgG antibody levels were lower in the vulnerable than healthy subjects (SMD = −1.62, 95% CI [−3.00, −0.23]) included in four studies (Palich et al., 2021b; Pimpinelli et al., 2021; Speer et al., 2021) with ≤21 days between the first vaccine dose and COVID-19 Ab testing, as well as in one study (Goupil et al., 2021), including 71 vulnerable and 20 healthy subjects, with >21 days between the first vaccine and testing (SMD = −1.88, 95% CI [−2.45, −1.31]). IgG antibody levels were also lower in vulnerable than healthy subjects included in nine studies (Grupper et al., 2021a; Grupper et al., 2021b; Geisen et al., 2021; Herzog Tzarfati et al., 2021; Korth et al., 2021; Pimpinelli et al., 2021; Rabinowich et al., 2021; Speer et al., 2021) in which ≤21 days elapsed between the second vaccine dose and COVID-19 Ab testing (SMD = −2.71, 95% CI [−3.77, −1.65]). Similar results were seen in three studies (Massarweh et al., 2021; Moor et al., 2021; Yanay et al., 2021), including 358 vulnerable and 239 healthy subjects with >21 days between the second vaccine dose and Ab testing (SMD = −0.98, 95% CI [−1.16, −0.81]) (Table 3).

#### 3.7.6 Antibody detection methods

Four studies (Geisen et al., 2021; Goupil et al., 2021; Moor et al., 2021; Speer et al., 2021), including 210 vulnerable and 137 healthy subjects, used ELISA to measure the humoral immune response. IgG antibody levels were lower in the vulnerable than in the healthy subjects (SMD = −1.14, 95% CI [−1.67, −0.61]). Ten studies (Grupper et al., 2021a; Grupper et al., 2021b; Palich et al., 2021b; Herzog Tzarfati et al., 2021; Korth et al., 2021; Massarweh et al., 2021; Pimpinelli et al., 2021; Rabinowich et al., 2021; Yanay et al., 2021), including 1,028 vulnerable and 583 healthy subjects, used LIAISON to test antibody levels and the vulnerable subjects had lower IgG antibody levels than the healthy subjects (SMD = −2.47, 95% CI [−3.25, −1.68]) (Table 3).

#### 3.7.7 Antibody type

Nineteen experiments were performed to measure serum anti-S, anti-S1, and anti-RBD IgG antibody levels. Eleven datasets (Grupper et al., 2021a; Palich et al., 2021b; Geisen et al., 2021; Herzog Tzarfati et al., 2021; Korth et al., 2021; Massarweh et al., 2021; Moor et al., 2021; Pimpinelli et al., 2021; Rabinowich et al., 2021; Yanay et al., 2021) with anti-S antibody data (SMD = −2.01, 95% CI [−2.66, −1.37]) and three datasets (Grupper et al., 2021b; Palich et al., 2021b; Speer et al., 2021) with anti-S1 antibody data (SMD = −1.84, 95% CI [−3.53, −0.15]) had lower antibody levels in the vulnerable than in the healthy group. In one experiment (Goupil et al., 2021) measuring anti-RBD antibodies, including 71 vulnerable and 20 healthy people, IgG antibody levels were lower in the vulnerable than in the healthy group (SMD = −1.88, 95% CI [−2.45, −1.31]) (Table 3).

#### 3.7.8 COVID-19 infection after vaccination

Vulnerable individuals who were uninfected with COVID-19 (SMD = −1.30, 95% CI [−1.91, −0.70]) (Palich et al., 2021a; Geisen et al., 2021; Massarweh et al., 2021; Pimpinelli et al., 2021; Rabinowich et al., 2021; Tzioufas et al., 2021), had infection after vaccination (SMD = −1.84, 95% CI [−3.09–0.60]) (Grupper et al., 2021b; Goupil et al., 2021; Yanay et al., 2021), or were uncertain about infection (SMD = −2.99, 95% CI [−5.16, −0.82]) (Grupper et al., 2021a; Korth et al., 2021; Moor et al., 2021), had lower IgG antibody levels than healthy individuals.

## 4 Discussion

Vulnerable populations are often more prone to severe symptoms or adverse outcomes of COVID-19 infection and have lower seroconversion rates after COVID-19 vaccination than healthy populations due to a depressed immune response. Thus, more attention is needed to prevent COVID-19 prevention in these populations using tailored vaccination regimens. This study conducted a meta-analysis of virus-specific antibody measurements and the proportion of vulnerable and healthy individuals with serum SARS-CoV-2 antibodies following COVID-19 vaccination was assessed to evaluate the effectiveness and safety of the vaccine in at-risk populations. The findings indicated that vulnerable individuals had lower virus-specific antibody levels after vaccination than healthy individuals. However, both groups had a similar incidence of adverse events, and antibody-positive subjects in either group were less likely to develop severe COVID-19 infection. Thus, while vulnerable populations were less likely to seroconvert after COVID-19 vaccination, the vaccine remained effective and safe and there was no association between vaccination and the development of adverse reactions in at-risk individuals.

IgG appears earlier and is more sensitive and easily detected than IgM after COVID-19 infection. As a result, most studies evaluate seroconversion by detecting IgG positivity (Lee et al., 2020). However, one study (Chen et al., 2021) found that the most accurate test for determining whether a subject is COVID-19 positive is assessing IgM or IgG positivity. Based on all serological results, measuring IgA antibody production and positive detection rates were superior to those of the other tests. Yu et al. (Yu et al., 2020) found that IgA positivity has high sensitivity but poor specificity and can only be used as a reference for positive patients, indicating the need for additional evaluation. Lee et al. (Chen et al., 2021) suggested that the most effective vaccine regimen for vulnerable populations should be developed by combining IgG, IgM, and IgA positivity rates.

To identify the potential factors that influence IgG antibody production, subgroup analyses were performed. All vulnerable populations had lower IgG antibody production than healthy populations after COVID-19 vaccination, with solid cancer patients having the highest IgG antibody levels of all vulnerable populations, hematological cancer patients having the lowest, and hemodialysis patients having higher IgG antibody levels than hematological cancer patients. Low antibody production in hematological cancer patients may be explained by the strong suppression of humoral and cellular immune capacity caused by both the treatment and underlying disease (Espi et al., 2022). The use of monoclonal antibodies in hematological cancer patients and hemodialysis patients could be used to supplement the relatively low seroconversion levels observed after vaccination.

In subjects who received BNT162b2 alone or multiple vaccine types (Pfizer-BioNTech BNT162b2 and Moderna mRNA-1273), IgG antibody levels were weaker in the vulnerable population. There was moderate variation in antibody production, reflecting the different responses elicited by a combination vaccine versus a single mRNA vaccine in vulnerable patients. However, the measurement of a single seroconversion rate is not sufficient to indicate that the combined vaccine is superior to a single vaccine. More data are required to recommend the use of a combined vaccine to improve seroconversion in vulnerable populations. After either one or two COVID-19 vaccine doses, the vulnerable population had lower IgG antibody levels than the healthy population. This pattern of poorer vaccination outcomes in vulnerable populations is also seen following other types of vaccine, likely because of the inferior immune capacity of these patients (Osmanodja et al., 2022). However, some studies (Lisboa Bastos et al., 2020; Gounant et al., 2022) indicate that more vaccine doses can increase antibody production in cancer patients and should be considered to achieve seroconversion levels comparable to healthy individuals. IgG antibody levels in vulnerable populations were more varied when the interval between COVID-19 vaccine doses was ≤21 days and were elevated when the interval was >21 days. This may be because the immune system is able to produce more antibodies in response to vaccination at intervals >21 days. More data are needed to determine the optimal interval between vaccine doses in vulnerable populations.

Post-vaccination blood testing using either an ELISA or LIAISON showed that the vulnerable population had lower IgG antibody levels than the healthy population. IgG antibody levels were elevated when serological testing was performed ≥21 days after both doses. This may be due to a delayed immune response to the vaccine in vulnerable populations, with increased antibody production occurring after 21 days. Thus, testing for antibodies 21 days after vaccination may be considered for future testing of vulnerable populations. An ELISA was able to detect higher antibody levels than LIAISON. This may be due to the greater sensitivity of ELISA for anti-RBD antibodies (Lisboa Bastos et al., 2020) and suggests that ELISA testing for anti-RBD antibodies should be considered for future testing of vulnerable populations. Vulnerable populations had lower levels of anti-S, anti-S1, and anti-RBD IgG antibodies than healthy populations. Anti-RBD antibodies cause greater virus neutralization than anti-spike-in antibodies, however, more studies are needed to assess the effectiveness of COVID-19 vaccination in vulnerable populations by detecting anti-RBD antibodies.

Due to the high infectivity of SARS-CoV-2, subjects may become infected after receiving the COVID-19 vaccine. Studies indicate that post-vaccination infection can lead to higher serum IgG antibody levels. Individuals who become infected from the vaccine may artificially lead to high IgG antibody levels. The current study found that IgG antibody levels in vulnerable patients were lower than in healthy subjects after vaccination, regardless of the presence of disease. While IgG antibody levels were elevated in a subgroup of subjects with COVID-19 infection after vaccination, they remained low compared to the healthy population. These findings suggested that COVID-19 infection after vaccination has a limited impact on the evaluation of vaccine efficacy.

Serum antibody levels are lower after COVID-19 vaccination than after infection with the virus (Rastawicki and Płaza, 2021). This suggests that antibody production may not be sufficient to completely protect healthy people from COVID-19 and indicates why the low serum antibody levels in vulnerable individuals often fail to protect them from COVID-19. These findings suggest that vulnerable populations must maintain a level of physical protection against SARS-CoV-2 after vaccination. However, vaccination can reduce the rate of COVID-19 infection and effectively lower the occurrence of severe respiratory symptoms in infected individuals. While vaccination is not likely to prevent COVID-19 infection in vulnerable individuals, it can prevent the occurrence of acute and severe respiratory diseases, thereby reducing mortality.

In this study, the seroconversion rate was lower in the vulnerable than in the healthy population, regardless of whether the subject was infected with COVID-19 after vaccination, a finding consistent with other studies. A meta-analysis (Del Bello et al., 2022) found that while patients with organ transplants only had a 34% seroconversion rate after the second COVID-19 vaccine dose, patients receiving a third dose had a seroconversion rate of 66%. However, those who did not seroconvert after the second dose continued to have a negative seroconversion rate after the third dose. Studies suggest improving vaccination regimens for organ transplant patients using monoclonal antibodies to supplement or replace those produced by vaccination. Other studies (Piechotta et al., 2022) have shown that the post-vaccination humoral response is lower in patients with hematological cancers than in healthy individuals, with chronic lymphocytic leukemia patients having the lowest response. Patients with impaired B lymphocytes have poor immunogenicity even after a third booster dose. Thus, depressed seroconversion rates after COVID-19 vaccination are unavoidable in vulnerable populations and a third booster dose can have a mitigating though somewhat limited impact on antibody production. For patients who continue to have depressed seroconversion rates after a third booster dose, monoclonal antibodies are recommended as a supplement. The use of monoclonal antibodies instead of a third booster dose could also be considered, however, the efficacy of this regimen requires further study.

This study has some limitations. First, no studies have been conducted to date on subjects vaccinated against each SARS-CoV-2 subtype. Since the virus has a very quick mutation rate and each subtype has a different level of virulence and infectivity, protection of the COVID-19 vaccine against vulnerable populations may differ. Second, most subjects included in this meta-analysis received the BNT162b2 COVID-19 vaccine and, to a lesser extent, the Moderna mRNA-1273 vaccine. Current data on vulnerable populations vaccinated with AD26.COV2.S, AZD-1222/ChAdOx1 nCoV-19, or other approved vaccines that met the inclusion criteria for this meta-analysis are scarce. Multiple future trials are required to further assess the efficacy and safety of these vaccines in vulnerable populations. Third, most of the studies included in this meta-analysis focused on the serum antibody levels of the subjects after vaccination and paid comparatively little attention to the cellular immune responses. To obtain more accurate results, further study of the post-vaccination cellular immune response in vulnerable populations is needed.

## 5 Conclusion

This meta-analysis found that post-COVID-19 vaccination seroconversion rates were generally poorer in vulnerable than in healthy populations, but there was no difference in adverse events. Patients with hematological cancers had the lowest IgG antibody levels of all vulnerable populations, and so more focus on these patients is recommended. Subjects who received the combined vaccine had higher antibody levels than those who received the single vaccine. Additional studies are needed to inform the development of more tailored vaccination protocols for vulnerable populations.

## Data Availability

The original contributions presented in the study are included in the article/Supplementary Material, further inquiries can be directed to the corresponding authors.
