# Supplementary material for: COVID-19 vaccination effectiveness and safety in vulnerable populations: a meta-analysis of 33 observational studies
Source: Front Pharmacol. 2023 Jun 23;14:1144824. doi: 10.3389/fphar.2023.1144824 (PMC10326898; doi:10.3389/fphar.2023.1144824)
Supplement: Supplementary file 1 [file DataSheet1.PDF]

Supplementary Method 1 Search Strategy

1. PubMed 1946 to July 12, 2022

#1 "Vulnerable Populations"[MeSH] OR "Vulnerable Population*"[tiab] OR "Vulnerable Patient*"[tiab] OR vulnerable[tiab] OR Sensitive Population*[tiab] OR Underserved Population*[tiab] OR Aged[MeSH] OR elderly[tiab] OR "older adults"[tiab] OR "Multiple Chronic Conditions"[MeSH] OR "chronic disease"[tiab] OR disadvantage*[tiab] "Immunocompromised Host"[MeSH] OR "Immunocompromised Patients"[tiab] OR "Organ Transplantation"[MeSH] OR Neoplasms[MeSH] OR Tumor[tiab] OR Cancer[tiab] OR Malignancy[tiab]

#2 "COVID-19 Vaccines"[MeSH] OR "COVID 19 Vaccines"[tiab] OR "COVID 19 Virus Vaccine"[tiab] OR "COVID19 Vaccine"[tiab] OR "SARS-CoV-2 Vaccine"[tiab] OR "SARS CoV 2 Vaccine"[tiab] OR "SARS2 Vaccine"[tiab] OR "Coronavirus Disease 2019 Vaccine"[tiab] OR "Coronavirus Disease 2019 Virus Vaccine"[tiab] OR "Coronavirus Disease 19 Vaccine"[tiab] OR "COVID 19 Vaccine"[tiab] OR "2019-nCoV Vaccine"[tiab] OR "2019 Novel Coronavirus Vaccine"[tiab] OR "2019 nCoV Vaccines"[tiab] OR "COVID-19 Vaccine"[tiab] OR "SARS Coronavirus 2 Vaccines"[tiab]

#3 #1 AND #2

2. EMbase 1974 to July 12, 2022

#1 ‘Vulnerable Populations’/exp OR ‘Vulnerable Population*’:ab,ti OR ‘Vulnerable Patient*’:ab,ti OR vulnerable:ab,ti OR ‘Sensitive Population*’:ab,ti OR ‘Underserved Population*’:ab,ti OR Aged/exp OR elderly:ab,ti OR ‘older adults’:ab,ti OR ‘Multiple Chronic Conditions’/exp OR ‘chronic disease’:ab,ti OR disadvantage*:ab,ti OR ‘Immunocompromised Host’/exp OR ‘Immunocompromised Patients’:ab,ti OR ‘Organ Transplantation’/exp OR Neoplasms/exp OR Tumor:ab,ti OR Cancer:ab,ti OR Malignancy:ab,ti

#2 COVID-19 Vaccines/exp OR "COVID 19 Vaccines":ab,ti OR "COVID 19 Virus Vaccine":ab,ti OR "COVID19 Vaccine":ab,ti OR "SARS-CoV-2 Vaccine":ab,ti OR "SARS CoV 2 Vaccine":ab,ti OR "SARS2 Vaccine":ab,ti OR "Coronavirus Disease 2019 Vaccine":ab,ti OR "Coronavirus Disease 2019 Virus Vaccine":ab,ti OR "Coronavirus Disease 19 Vaccine":ab,ti OR "COVID 19 Vaccine":ab,ti OR "2019-nCoV Vaccine":ab,ti OR "2019 Novel Coronavirus Vaccine":ab,ti OR "2019 nCoV Vaccines":ab,ti OR "COVID-19 Vaccine":ab,ti OR "SARS Coronavirus 2 Vaccines":ab,ti

#3 #1 AND #2 AND [english]/lim AND ([adult]/lim OR [young adult]/lim OR [middle aged]/lim OR [aged]/lim OR [very elderly]/lim) AND [humans]/lim

3. Cochrane Central Register of Controlled Trials, Issue 6 of 12, 2022

#1 MeSH descriptor: [Vulnerable Populations] explode all trees
#2 MeSH descriptor: [Aged] explode all trees
#3 MeSH descriptor: [Chronic Disease] explode all trees
#4 MeSH descriptor: [Immunocompromised Host] explode all trees
#5 MeSH descriptor: [Organ Transplantation] explode all trees
#6 MeSH descriptor: [Neoplasms] explode all trees
#7 ("Vulnerable Population*" OR "Vulnerable Patient*" OR vulnerable OR Sensitive Population* OR Sensitive Population Group* OR Underserved Population* OR Underserved Patient* OR elderly OR "older adults" OR "chronic disease" OR disadvantage* OR "Immunocompromised Patients" OR Tumor OR Cancer OR Malignancy):ti,ab,kw
#8 #1 OR #2 OR #3 OR #4 OR #5 OR #6 OR #7
#9 MeSH descriptor: [COVID-19 Vaccines] explode all trees
#10 ("COVID 19 Vaccines" OR "COVID 19 Virus Vaccine" OR "COVID19 Vaccine" OR "SARS-CoV-2 Vaccine" OR "SARS CoV 2 Vaccine" OR "SARS2 Vaccine" OR "Coronavirus Disease 2019 Vaccine" OR "Coronavirus Disease 2019 Virus Vaccine" OR "Coronavirus Disease 19 Vaccine" OR "COVID 19 Vaccine" OR "2019-nCoV Vaccine" OR "2019 Novel Coronavirus Vaccine" OR "2019 nCoV Vaccines" OR "COVID-19 Vaccine" OR "SARS Coronavirus 2 Vaccines"):ti,ab,kw
#11 #9 OR #10
#12 #8 AND #11
